# miR-107 Inhibits the Proliferation of Gastric Cancer Cells *In vivo* and *In vitro* by Targeting TRIAP1

**DOI:** 10.3389/fgene.2022.855355

**Published:** 2022-04-11

**Authors:** Jiexin Yan, Lu Dai, Jun Yuan, Min Pang, Yueqiu Wang, Lang Lin, Yawei Shi, Fuli Wu, Rongping Nie, Qiuling Chen, Lei Wang

**Affiliations:** ^1^ Emergency of Department, Changhai Hospital, Shanghai, China; ^2^ The Fourth Outpatient Department, The Affiliated Stomatological Hospital of Nanjing Medical University, Nanjing, China; ^3^ Department of Oral and Maxilliofacial Surgery, The Affiliated Stomatological Hospital of Nanjing Medical University, Nanjing, China; ^4^ The Seventh Outpatient Department, The Affiliated Stomatological Hospital of Nanjing Medical University, Nanjing, China; ^5^ Cangnan Hospital Affiliated to Wenzhou Medical University, Wenzhou, China; ^6^ Department of Gastroenterology, Changhai Hospital, Shanghai, China

**Keywords:** gastric cancer, short non-coding RNA, cell proliferation, cell invasion, TRIAP1

## Abstract

Gastric cancer is a kind of gastrointestinal tumor with high morbidity and mortality. Finding effective methods for early diagnosis and treatment of gastric cancer has important significance and application prospects. MicroRNAs without protein coding potential affect the occurrence and development of gastric cancer. This study aims to explore the biological function and mechanism of microRNA-107 (miR-107) in gastric cancer. The results show that miR-107 is low expressed in gastric cancer, while TRIAP1 is highly expressed; the overexpression of miR-107 can inhibit the progression of gastric cancer *in vivo* and *in vitro*, while the overexpression plasmid of TRIAP1 can restore the miR-107 mimic-induced cell proliferation and metastasis inhibition, and the small interfering RNA of TRIAP1 can inhibit the cell proliferation and invasion induced by miR-107 inhibitor. In conclusion, the results of this study show that miR-107 can inhibit the proliferation of gastric cancer *in vivo* and *in vitro* by targeting TRIAP1.

## Introduction

Gastric cancer (GC) is a common malignant tumor with a high recurrence index and a high recurrence rate, with a mortality rate as high as 40% (Hurwitz et al., 2020). Although great progress has been made in the treatment of GC, the 5-year survival rate of patients diagnosed with metastatic GC is less than 20% (Anauate et al., 2017; Zhang et al., 2021), and the in-depth study of the pathogenesis of GC, new molecular markers and therapeutic targets are of great significance to its diagnosis and treatment.

MicroRNAs (miRNAs) are RNAs with a length of 18–24 nucleotides. They inhibit post-transcriptional protein translation by directly degrading mRNA or by binding to the 3′-untranslated region (3′-UTR) of target mRNA, and widely involved in cell development, differentiation, apoptosis, proliferation, metastasis and metabolism (Li et al., 2021). Recent studies have pointed out (Ai et al., 2018; Zhang et al., 2019; Wei et al., 2020a; Yu et al., 2020a) that miRNA is dysregulated in a variety of human cancers, and can be used as tumor promoters or suppressor genes to participate in tumor progression and occurrence. According to reports, miR-107 is differentially expressed in several types of cancers, such as breast cancer and colorectal cancer. In addition, in recent studies (Chen et al., 2019; Parvaee et al., 2019; Wang et al., 2019; Wei et al., 2020b; Zhang et al., 2020), miR-107 can promote the growth of pancreatic cancer cells; in breast cancer, it promotes the proliferation of cancer cells by targeting JAK2; in glioma and colon cancer, miR-107 promotes cancer cell proliferation and migration by targeting the CTGF-EGFR signaling pathway; in cervical cancer cells, miR-107 inhibits their migration and invasion by targeting the transcription factor SPI, by targeting Hippo Signal transducer YAP, miR-107 can inhibit the malignant progression of tumors in liver cancer cells; but its role in GC and its molecular mechanism have not been reported. In this context, this study detected the expression of miR-107 in four kinds of gastric cancer and normal gastric mucosal epithelial cells and further study its biological function and corresponding molecular mechanism.

## Materials and Methods

### GC Patient and Tissue Sample Collection

From June 2020 to August 2021, we collected tumors and adjacent tissues from 12 patients with gastric cancer in the Gastroenterology Department of our hospital. No patients received radiotherapy or chemotherapy before surgery, including 6 males and 6 females, 7 cases aged ≥ 60 years old, 6 cases aged < 60 years old, the specimens were stored in -196°C liquid nitrogen until western blot analysis and PCR detection. The use of specimens and research procedures have been approved by the hospital medical ethics committee (K202002146), and the patient’s informed consent was obtained.

### Experimental Materials and Instruments

Rabbit anti-mouse TRIAP1 monoclonal antibody (ab182858), rabbit anti-mouse GAPDH monoclonal antibody (ab8245), horseradish peroxidase labeled immunoglobulin conjugate-rabbit anti-mouse IgG conjugate (ab233006) were purchased from Abcam (United Kingdom); SuperScript IV Reverse Transcriptase (M614272), SuperScript IV One-Step RT-PCR Kit (M33253), SuperScript IV CellsDirect cDNA Synthesis Kit (M39128), RNase Inhibitor (M712192), TRIZOL (M33253), cMTT (M2003), Annexin V-FITC apoptosis detection kit (M2118), MEM (M8042), RPMI-1640 medium (R8758), FBS (F8687) were purchased from Thermo Fisher Scientific. CO_2_ incubator (Shanghai Boxun, cat: BC-J160S), 4°C centrifuge (eppendorf, centrifuge 5415R), PCR machine (eppendorfrealplex), ultra-clean workbench (Shanghai Boxun model SW-CJ-2FD), inverted fluorescence electron microscope (Leica DMI3000B), electrophoresis instrument (Tanon EPS300), GloMax^®^ Discover microplate reader (Promega, United States), Olympus inverted microscope GX41 (Olympus, Japan), CytoFLEX_ flow cytometer (Baker Mancourt, United States).

### Cell Culture

Culture human GC cell lines NCI-N87, CRL-5822, BGC-823 and normal gastric mucosal epithelial cells GES-1 in RPMI-1640 medium supplemented with 15% fetal bovine serum, 100 U/ml penicillin and 100 U/ml streptomycin, and place it in a humidified incubator at 37°C and 5% CO_2_.

### Cell Transfection and Grouping

The coding sequence of TRIAP1 was amplified by RT-PCR, and then cloned into the BstB I and Xba I sites of pCDH-EF1-MCS-T2A-Puro, and the pCD-EF1-MCS-T2A-Puro-TRIAP1 recombinant vector was transiently transfected into NCI-N87 cells to overexpress TRIAP1 and NCI-N87 cells transfected with an empty vector were used as controls. TRIAP1 small molecule interference siRNA was transfected to knock down TRIAP1 in NCI-N87 cells, and nonsense siRNA was transfected as a negative control (NC). siRNA NC, mimics NC, miR-NC mimics and inhibitors are synthesized by GenePharma (China). Inoculate NCI-N87 cells in 37°C, 5% CO_2_ environment, and add 15% FBS to RPMI-1640 medium. Using lipofectamine™2000 to transfer interfere siRNA of TRIAP1,TRIAP1overexpression plasmid, miR-107 mimic and miR-107 inhibitor according to the reagent manufacturer’s instructions.

Divide the cells into nine groups: Control group (DMSO), miR-107 mimics group (miR-107 mimics), miR-107 inhibitor group (miR-107 inhibitor), NC inhibitor + mimics (NC inhibitor + NC mimics), Si-TRIAP1 group (si-TRIAP1), TRIAP1Vector group (TRIAP1Vector), NC si + Vector group (nonsense siRNA + blank plasmid), mimics + Vector group (miR-107 mimics + TRIAP1Vector), NC mimics + NC Vector group (NC mimics + NC Vector), analysis of cell proliferation, apoptosis and metastasis activity *in vitro* after 24 h of different transfection.

### Real-Time Quantitative Polymerase Chain Reaction (qRT-PCR)

According to the instructions, use Trizol™ reagent to extract total RNA from cells, quantify RNA concentration by NanoDrop 2000 spectrometer, use RT/RI enzyme mixture and gDNA kit for cDNA reverse transcription, use TransStart^®^ Tip Green qPCR SuperMix (ROX) kit for qRT-PCR analysis, U6/GAPDH was used as an internal reference for TRIAP1 and miR-107. The primer sequences are shown in Table 1. TRIAP1 and miR-107 primers were synthesized by RiboBio Company. The 2^-△△CT^ method was used to quantify the relative intensities of TRIAP1 and miR-107 in different cells.

**TABLE 1:** Primer sequence used in PCR.

Gene	Forward (5′, -3′)	Reverse (5′, -3′)
miR-107	GAGGCCTCTGATGAATGA	TCCGATGGGACACAGTAT
TRIAP1	TAC​TTG​TAC​CCC​CCG​CGA​G	TTT​CTG​CGC​GAA​TCT​GTT​TG
miR-107 mimic	AUU​AUG​ACA​CCA​UUU​UGG​CA	UAA​UAC​UGU​CUG​GUA​AAA​CCG​U
miR-107inhibitor	ACG​GUU​UUA​CCA​GAC​AGU​AUU​A	
GAPDH	TTC​CAC​CCA​TGG​CAA​ATT​CC	ATC​TCG​CTC​CTG​GAA​GAT​GG

### Western Blot Assay

The gastric cancer and adjacent tissue samples were washed with PBS several times and broken into fragments less than 1mm3. The transfected NCI-N87 cell pellet was suspended in 50 μl RIPA buffer and lysed on ice for 30 min at 4°C. The supernatant protein was collected by centrifugation at 12,000 rpm for 15 min, and the protein concentration was measured by Bradford assay. The same amount of protein in each group was boiled for 5 min and separated on a 10% SDS-PAGE gel. The protein bands were transferred to the nitrocellulose membrane Above, block with TBST (50 mM Tris, 100 mM NaCl and 0.1% Tween-20, pH) containing 5% (w/v) skimmed milk, and with the primary antibodies TRIAP1 and GAPDH at 4°C overnight, and then with HRP-conjugated Affinipure goat anti-rabbit IgG was incubated for 2 h at room temperature. The protein bands were visualized by ECL reagent, and imaged and quantified using the Bio-Rad ChemiDoc™ MP system.

### Dual Luciferase Reporter Gene

Construct a wild-type or mutant plasmid at TRIAP1-3′-UTR site that binds to miR-107 for dual-luciferase reporter gene analysis, amplify and insert the psiCHECK™2.0 dual-luciferase vector containing XhoI and NotI, the recombinant expression vector of wild-type TRIAP1-3′-UTR is named psiCHECK-2-TRIAP1-3′-UTR-WT, and for mutant TRIAP1-3′-UTR, it is psiCHECK-2-TRIAP1-3′-UTR -MUT. Suspend 5×10^4^ NCI-N87 cells in 1 ml of RPMI-1640 containing 15% FBS, inoculate them into 6-well plate wells, and incubate them at 37°C and 5% CO2 for 24 h. Then, each group of NCI-N87 The cells were co-transfected with 2 μg psiCHECK-2-TRIAP1-3′-UTR-WT and MUT, which contained 25 μM miR-107 mimics or negative control, in the presence of 4 μl lipofectamine™2000, the transfection was carried out at 37°C and 5% CO_2_ for 8 h. After the culture medium was discarded, the culture was incubated for 16 h, centrifuged at 1,000 rpm for 5 min to collect the sediment of each group, and Lysis in 200 μl lysis buffer at 4°C for 15 min. Finally, put 20 μl of the lysate supernatant of each group into the luminometer tube, first mix it with 100 μl Luciferase Assay Reagent II, and then mix with 100 μlStop&Glo Reagent and use Dual-Luciferase reporter gene assay system to detect luciferase activity.

### MTT

Inoculate each group of cells (5×10^3^ cells) into a 96-well plate, and incubate at 37°C and 5% CO2 for 24, 48, 72 and 96 h. Add 0.5 mg/ml MTT (3-(4,5-Dimethylthiazole-2)-2,5-diphenyltetrazolium bromide) solution to each well of cells, incubate in the dark at 37°C and 5% CO_2_ for 4 h. After removing the supernatant, add 150 μl of DMSO to each well to dissolve the formazan, and microplate reader was used to measure the absorbance at 490 nm for cell activity quantification.

### Transwell

The cells were plated in BioCoat Matrigel Invasion Chambers at a density of 1×10^5^ cells/well, the chambers were inserted into the wells of the 24-well plate, and incubated in RPMI 1640 medium containing 10% fetal calf serum. After 4 h, the invaded cells were fixed and stained with 0.1% crystal violet and the number of cells in five random fields was counted.

### Xenograft Tumor

Twenty 6-week-old BALB/c athymic nude mice (females, weighing 16–20 g) were purchased from Beijing Huafukang Biotechnology Co., Ltd. (Beijing, China) and were cultured in animal centers under pathogen-free conditions. All animal experiments are in compliance with the “Guidelines for the Care and Utilization of Laboratory Animals.” Suspend NCI-N87 cells in 100 ml phosphate buffer solution, and then subcutaneously inoculate them into the right abdomen of nude mice (5×10^6^ cells/mouse). The transplanted nude mice were randomly divided into two groups (8 small mice in each group). Rats), the Contorl (normal saline) and miR-107 mimics (miR-107 mimics) groups were injected once a day at a dose of 3 nmol per mouse and injected into the implanted tumor. Monitor the tumor volume (V) by measuring the length (L) and width (W) with a vernier caliper, and use formula V (cm^3^) = W^2^×L/^2^ to calculate. After 4 weeks, all experimental mice were euthanized, and all tumors were removed and Weigh and perform immunohistochemistry.

### Statistical Analysis

The data is expressed as the mean ± SD of three independent experiments. GraphPad Prism 6.0 (La Jolla, California, United States) was used for statistical analysis. The difference between the two groups was evaluated by Student’s *t* test analysis, When *p* < 0.05, the difference is statistically significant.

## Results

### MiR-107 is Down-Regulated and TRIAP1 is Up-Regulated in GC Tumors

**FIGURE 1:**
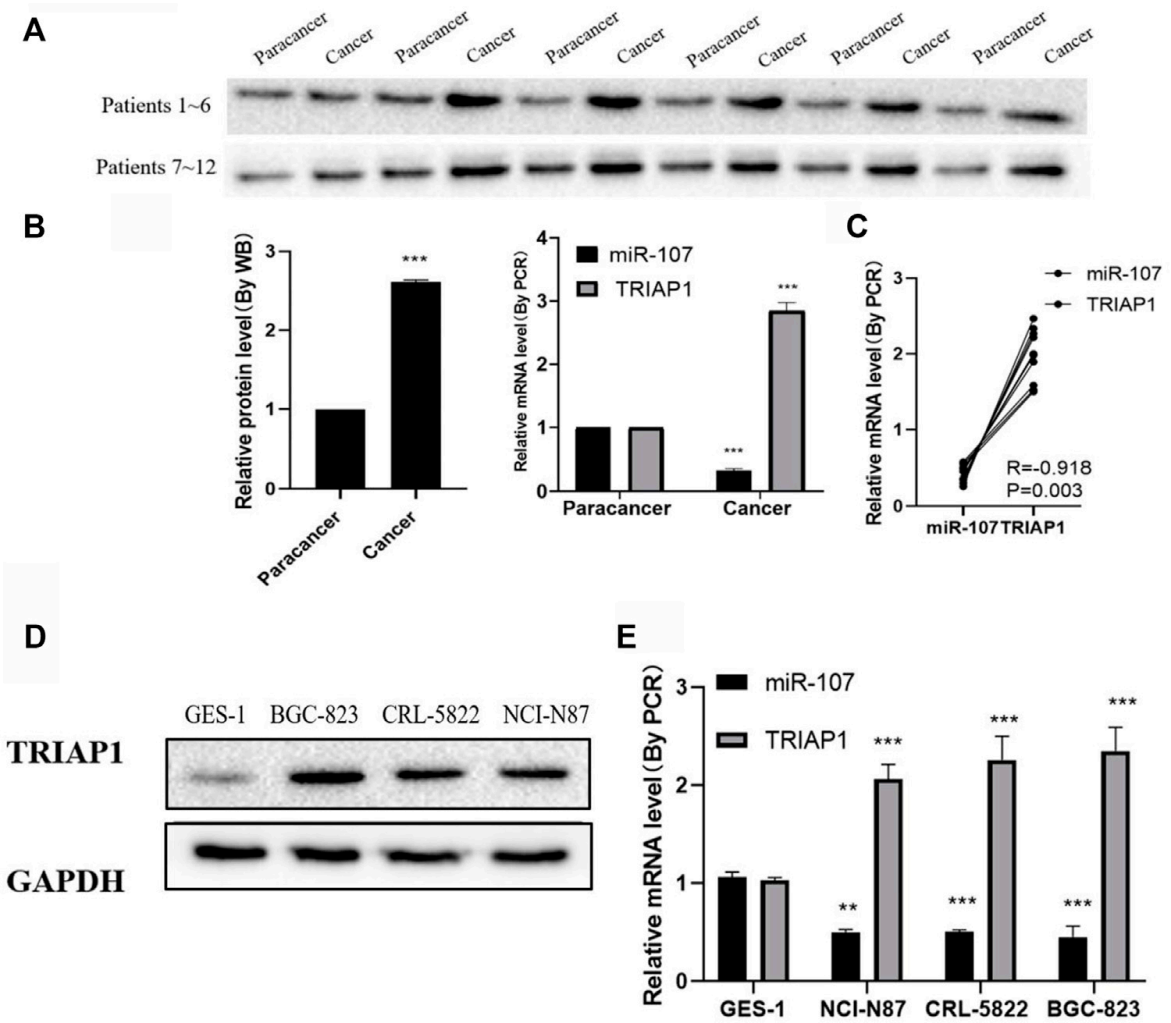
miR-107 is down-regulated and TRIAP1 is up-regulated in GC tumors **(A)** Western blot analysis of TRIAP1 in cancerous and adjacent tissues of patients with gastric cancer, **(B)** Quantitative analysis of RT-PCR and Western blotting, **(C)** Correlation analysis of the expression levels of TRIAP1 and miR-107 in the tumor tissues of GC patients, **(D)** Western blot analysis of TRIAP1, **(E)** RT-PCR quantitative analysis of TRIAP1. The data are expressed as the mean ± SD of three experiments. and the data is analyzed by Student t or ANOVA test, *p < 0.05, **p < 0.01, ***p < 0.001.

Compared with paracancer tissues, the levels of TRIAP1 protein and mRNA in the tumor tissues of patients were significantly increased (Figure 1A), while the levels of miR-107 were significantly reduced (Figure 1B), and the expression of miR-107 and TRIAP1 in tumor tissues of GC patients was negative correlated (R = −0.922, Figure 1C). Further *in vitro* experiments showed that compared with normal gastric epithelial mucosal cells GES-1, the expression level of TRIAP1 protein and mRNA significantly increased in human GC cell lines NCI-N87, CRL-5822 and BGC-823 cells (Figures 1D,E), while the expression level of miR-107 was significantly down-regulated (Figure 1E), due to the relatively high level of TRIAP1 protein in NCI-N87 cells, this cell line was used for later experiments.

### miR-107 Targets the Inhibition of TRIAP1 Expression

**FIGURE 2:**
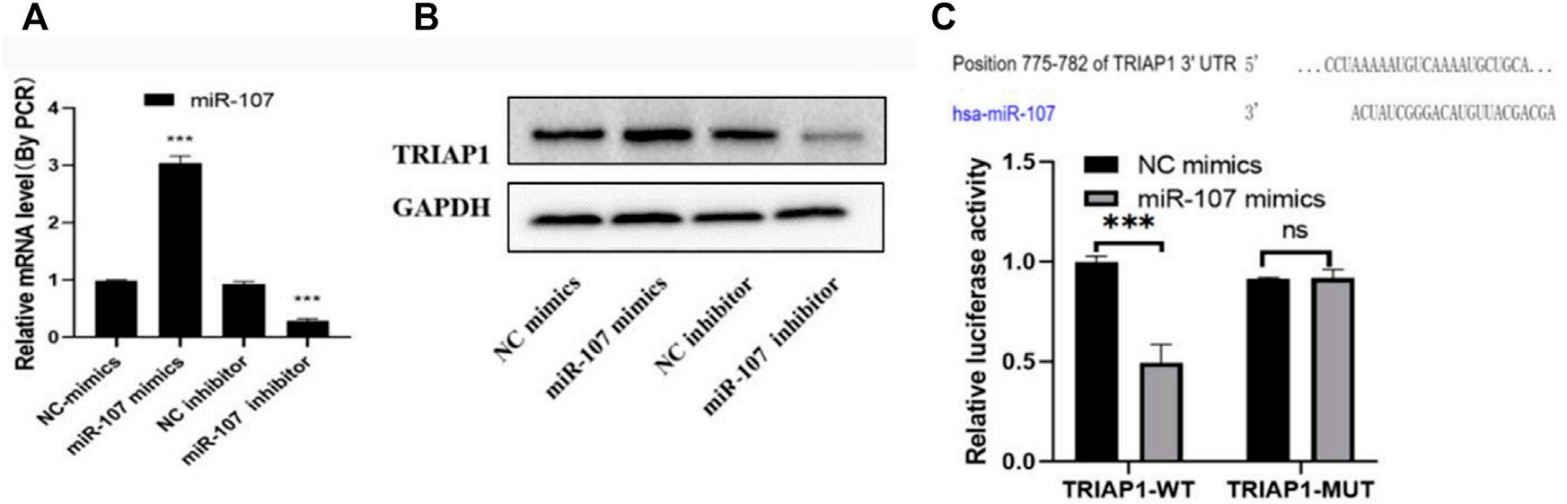
miR-107 negatively regulates TRIAP1 expression **(A)** The expression level of miR-107 was detected by RT-PCR after different transfections, **(B)** The protein level of TRIAP1 was detected by Western blot after different transfections, **(C)** The luciferase activity was detected by dual luciferase reporter gene after different transfections. The data is expressed as the mean ± SD of three experiments, and the data is analyzed by Student t or ANOVA test, *p < 0.05,**p < 0.01,***p < 0.001.

After miR-107 mimics transfection, the level of miR-107 mRNA was significantly increased, and the level of TRIAP1 protein was significantly reduced. After transfection of miR-107 inhibitor, the level of miR-107 mRNA in NCI-N87 cells was significantly decreased, and the level of TRIAP1 protein was significantly reduced. (Figures 2A,B), further dual-luciferase reporter gene detection showed that miR-107 mimics transfection can significantly inhibit the fluorescence of NCI-N87 cells transfected with psiCHECK-2-TRIAP1-3′-UTR-WT, and it has no significant effect on fluorescein activity in NCI-N87 cells transfected with psiCHECK-2-TRIAP1-3′-UTR-MUT (Figure 2C).

### TRIAP1 Promotes the *In vitro* Activity of NCI-N87 Cells

**FIGURE 3:**
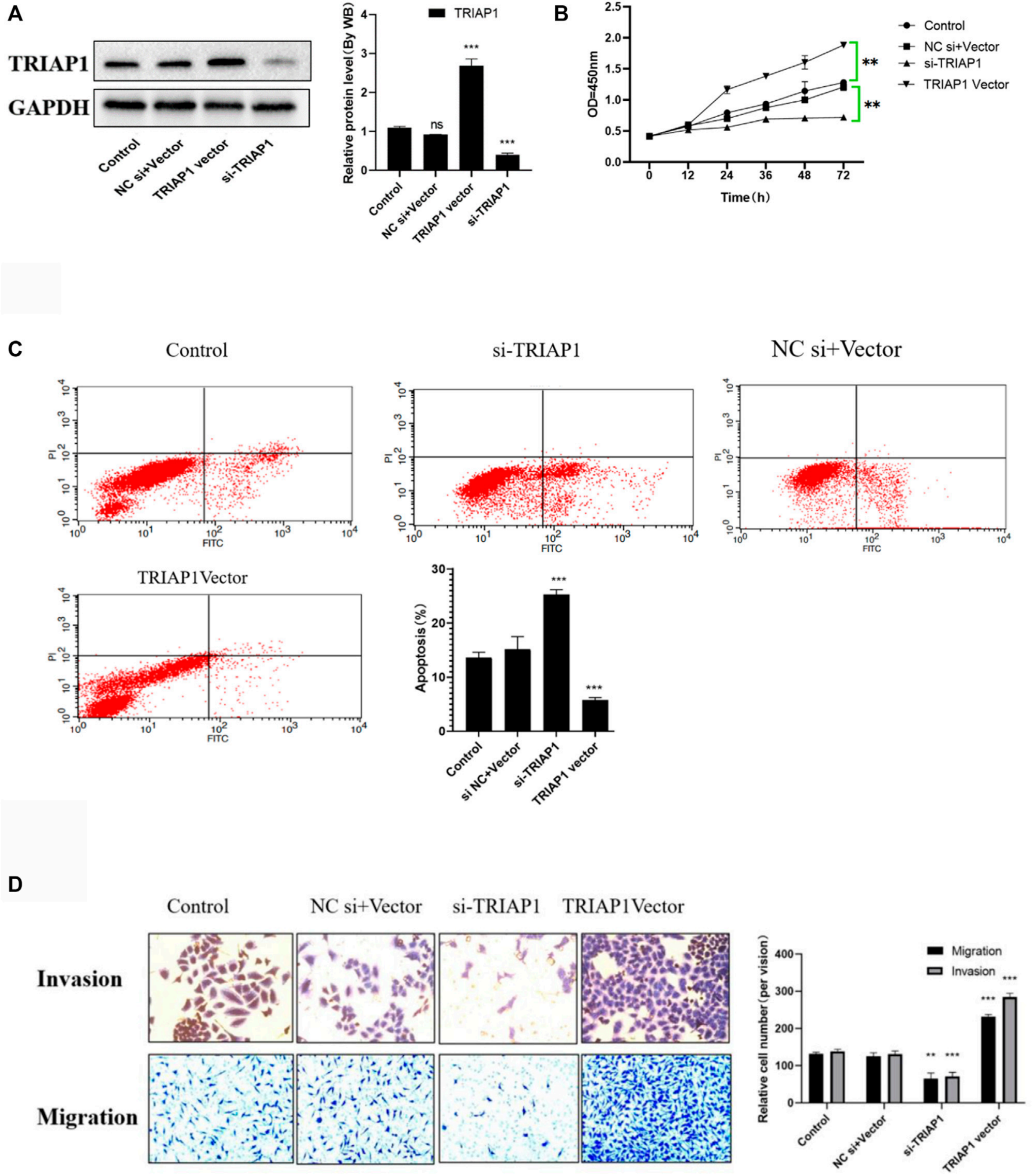
TRIAP1 promotes the activity of NCI-N87 cells *in vitro*. **(A)** The expression level of TRIAP1was detected by Western-blot after different transfections, **(B)** Proliferation activity of NCI-N87 cells after different transfection, **(C)** Apoptosis of NCI-N87 cells after different transfection, **(D)** Invasion and migration of NCI-N87 cells after different transfections was detected by Transwell, (original picture X100), Compared with the control group,*p < 0.05,**p < 0.01,***p < 0.001.

After transfection with si-TRIAP1 or TRIAP1 vector, the transfection efficiency was detected by Western blot. The results showed that TRIAP1 expression in NCI-N87 cells transfected with siTRIAP1 was significantly knocked down, while TRIAP1 expression was significantly up-regulated after TRIAP1 vector transfection (Figure 3A); Transwell and MTT analysis showed that compared with the control group after transfection with si-TRIAP1, cell migration and invasion capabilities were significantly reduced, proliferation activity was significantly inhibited, and apoptosis was significantly increased. Compared with the control plasmid, overexpression of TRIAP1 can significantly enhance the migration and invasion capabilities of NCI-N87 cells. Promote the proliferation activity of NCI-N87 cells and inhibit the apoptosis of NCI-N87 cells (Figures 3B–D).

### MiR-107 Inhibits the *In vitro* Activity of NCI-N87 Cells

**FIGURE 4:**
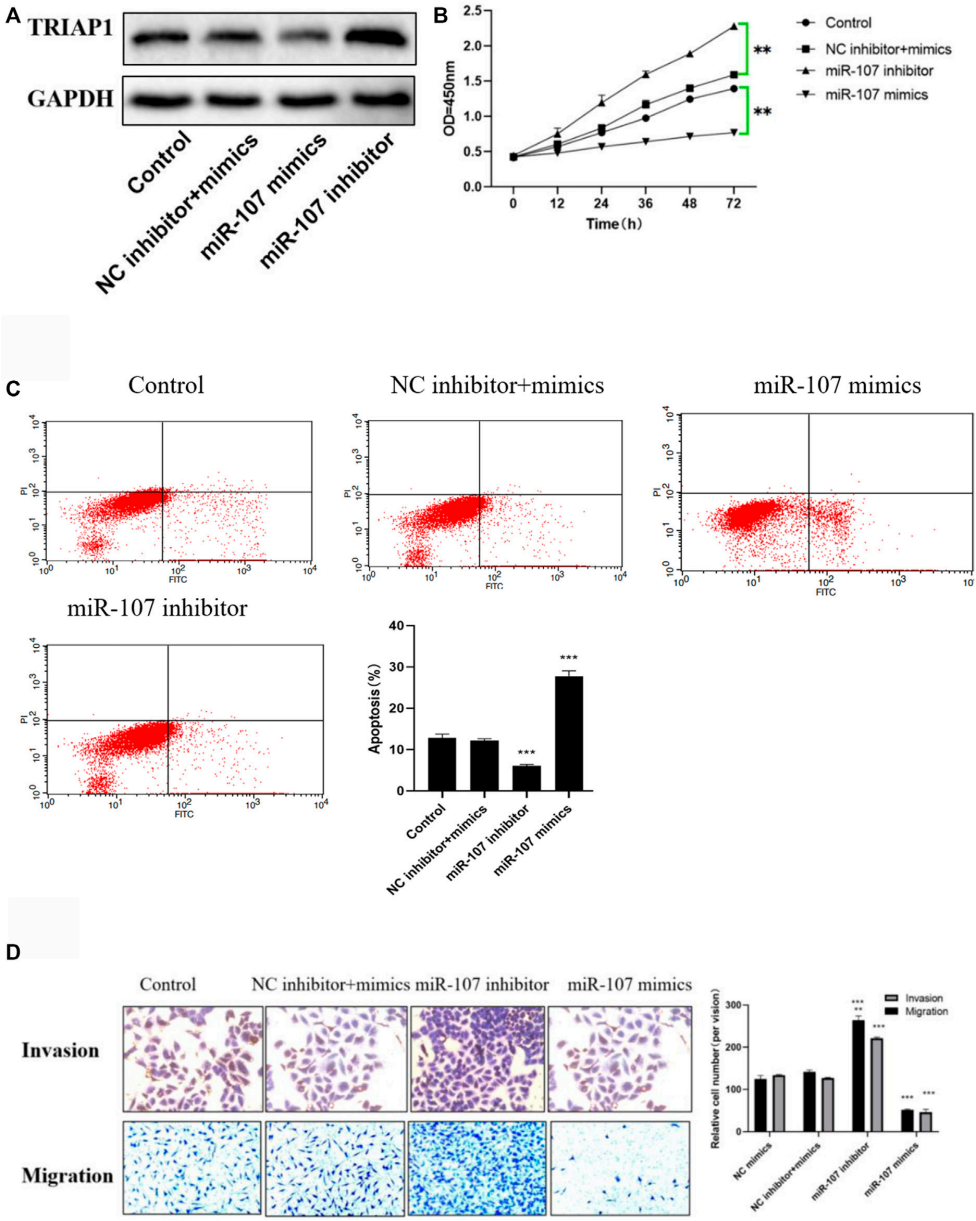
miR-107 inhibits metastasis and proliferation of NCI-N87 cells *in vitro* **(A)** Protein expression of TRIAP1 after miR-107 knock-down/overexpression, **(B)** Proliferation analysis of NCI-N87 cells after miR-107 knock-down/overexpression; **(C)** Apoptosis analysis of NCI-N87 cells after miR-107 knock-down/overexpression; **(D)** Invasion and migration of NCI-N87 cells after miR-107 knock-down/overexpression (original picture X100), Compared with the control group,*p < 0.05,**p < 0.01,***p < 0.001.

MTT and apoptosis analysis showed that compared with NCI-N87 cells in the control group or NC inhibitor + mimics group, after transfection with miR-107 mimics, cell proliferation was significantly reduced, and cell apoptosis was significantly increased. Further Transwell analysis showed that, compared with the NCI-N87 cells transfected with NC mimics, the miR-107 mimic transfection has significantly reduced cell migration and invasion capabilities (Figures 4A–D), while miR-107 inhibitor transfection significantly enhanced the migration and invasion ability of NCI- N87 cells, promotes cell proliferation activity and inhibits the apoptosis of NCI-N87 cells (Figures 4A–D).

### MiR-107 Mediates the *In vitro* Activity of NCI-N87 Through TRIAP1

**FIGURE 5:**
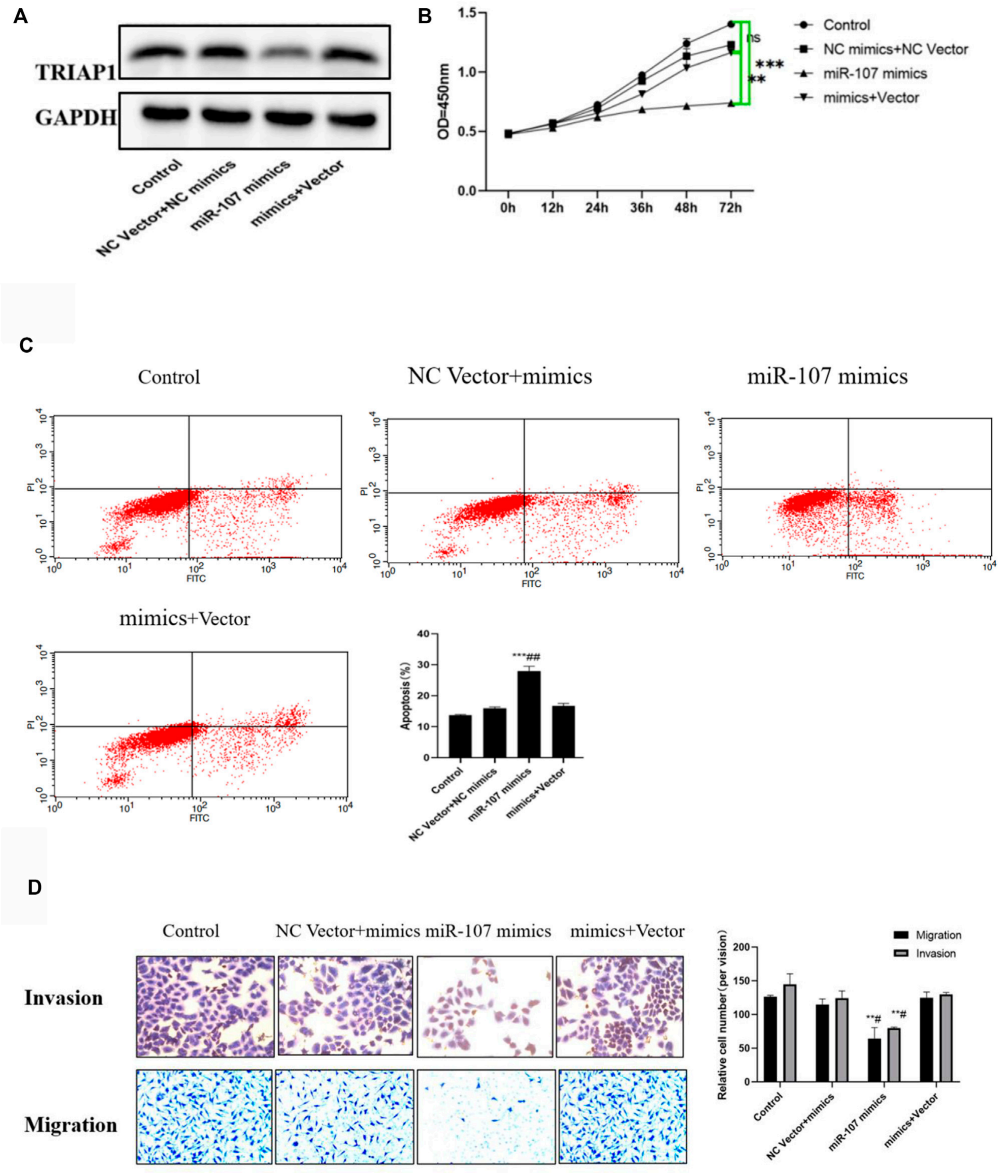
miR-107 mediates NCI-N87 transfer and proliferation through TRIAP1. **(A)** Western-blotting analysis and quantitation of TRIAP1 after different transfections, **(B)** Proliferation activity of NCI-N87 cells after different transfection, **(C)** Invasion and migration of NCI-N87 cells after different transfections, **(D)** Apoptosis of NCI-N87 cells after different transfection. Compared with the control group/NC Vector + mimics group, *p < 0.05, **p < 0.01, ***p < 0.001, compared with the miR-107 mimics group, #*p* < 0.05.

Compared with the miR-107 mimics group, overexpression of TRIAP1 in NCI-N87 cells can significantly promote the expression of TRIAP1, reverse the cell migration and invasion ability inhibited by miR-107 mimics, promote cell proliferation activity, and inhibit cell apoptosis induced by miR-107 mimics (Figures 5A–D).

### miR-107 Inhibits Tumor Progression *In vivo*

**FIGURE 6:**
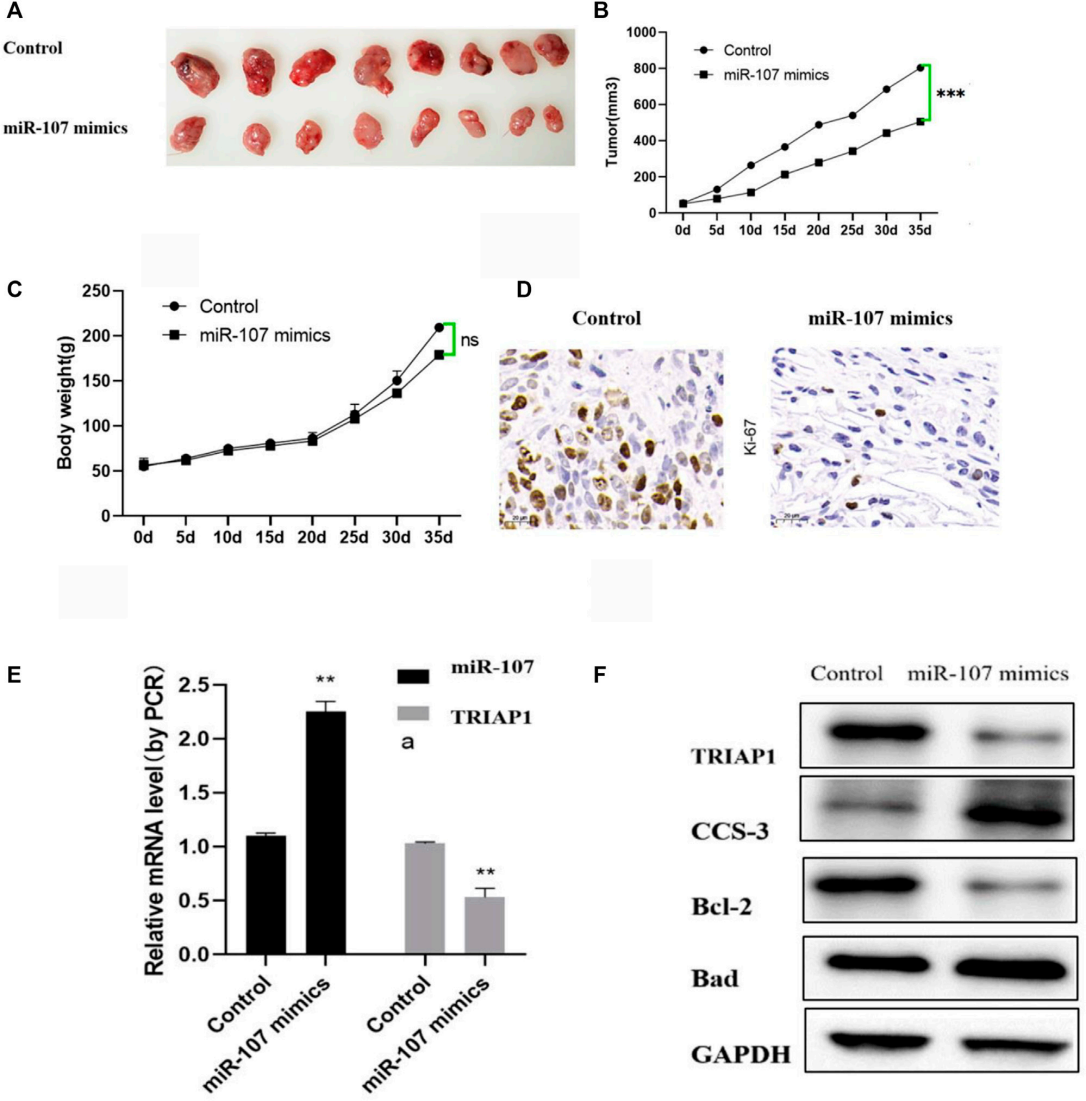
miR-107 inhibits tumor progression *in vivo.* **(A)** Representative pictures of tumors after different treatments, **(B)** Tumor progression after different treatments, **(C)** Mouse body weight changes after different treatments, **(D)** Immunohistochemical analysis of Ki-67 expression in tumor tissues (original Picture X100), **(E)** Expression levels of miR-107 and TRIAP1 in tumor tissue, **(F)** Expression levels of TRIAP1 and apoptosis-related proteins in tumor tissues, Compared with the control group, *p < 0.05,**p < 0.01, ***p < 0.001.

Compared with the mice in the control group, the tumor growth was significantly slower after miR-107 mimics treatment, the weight difference between the two groups of mice did not change significantly. Further immunohistochemical and western blot analysis showed that after miR-107 mock treatment, the expression of miR-107 in tumors was significantly increased, while the expression of TRIAP was significantly decreased, the expression of tumor growth marker Ki67 was significantly decreased, and the expression of apoptosis-related proteins was significantly (Bad, CCS-3) increased, suggesting that miR-107 could inhibit tumor progression in mice (Figures 6C–F).

## Discussion

Gastric cancer is an important cause of high mortality, and its incidence is still increasing. Although there are treatments for this type of cancer, the recurrence is frequent and the treatment has many side effects. Therefore, it is necessary to explore new drugs or determine specific therapeutic targets for the treatment of gastric cancer (Hurwitz et al., 2020; Zhang et al., 2021). Over the years, a series of miRNAs have been found to be dysregulated in a variety of cancer cells and are associated with cancer progression. And they are potential targets for cancer treatment. The results of this study show that miR-107 is significantly low in GC, the expression of miR-107 is obviously low in GC, while the expression of TRIAP1 is obviously up-regulated in GC. Further functional and mechanism analysis shows that miR-107 can exert its biological function by targeting TRIAP1, which can effectively inhibit GC progress *in vivo* and *in vitro*.

MiR-107 has a variety of biological activities, but its role in gastric cancer is still controversial. For example, Wei et al. (2020b) pointed out that circHIPK3 promotes the proliferation and migration of gastric cancer cells through the sponge miR-107 and the regulation of BDNF expression. Wang et al. (2019) pointed out that miR-107 activates PI3K-AKT signal by targeting FAT4 to promote the growth and metastasis of GC. In contrast, Chen et al. (2019) pointed out that lncRNA PCAT18 can promote the progression of gastric cancer by down-regulating the MiR-107/PTEN/PI3K/AKT signaling pathway. Cheng et al. (2018) confirmed that microRNA-107 inhibits the proliferation and metastasis of gastric cancer cells by targeting the PI3K/AKT pathway. Consistent with the studies of Chen P and Feng C, the results of this study confirmed that miR-107 is low expressed as a tumor suppressor gene in GC. *In vitro* function and mechanism analysis showed that miR-107 mimics can inhibit the proliferation of NCI-N87 cells *in vivo* and *in vitro*, and down-regulates the invasion and migration ability of NCI-N87 cells, while transfection with miR-107 inhibitor promotes the proliferation of NCI-N87 *in vivo* and *in vitro*, and up-regulates the invasion and migration of NCI-N87 cells at the same time. Wang et al. (2014) confirmed that 12 miRNAs including miR-107 are significantly dysregulated in GAC tissues by MiRNA microarray analysis, while sequencing of miR-107 promoters identified 3 SNPs (rs11185777, rs78591545 and rs78591545). rs2296616), the correlation analysis showed that the T allele of rs2296616 (T > C) was significantly associated with the reduction of the patient’s prognostic risk (TT vs. TC/CC, OR = 0.39, 95% CI = 0.31–0.49), The C allele is associated with an increase in the prognostic risk of patients (OR = 1.49, 95% CI = 1.01–2.20), which may explain the different biological effects of miR-107 in gastric cancer in this study, which may be attributed to the differences caused by sampling errors.

TRIAP1 (TP53-regulated apoptosis inhibitor 1, also known as P53CSV) contains 76 amino acids and is induced by TP53 under the drive of low genotoxicity. The level of TRIAP1 mRNA was detected in human tissues, and the results showed that this gene is also up-regulated in a variety of malignant tumors, and it plays a role in promoting cancer in most tumors. For example (Liu et al., 2021) miRNA-539 can inhibit the proliferation, migration and invasion of osteosarcoma cells by targeting TRIAP1, and promote the apoptosis of osteosarcoma cells. The lncRNA MFI2-AS1/miR-125a-5p axis up-regulates TRIAP1 to promote thyroid cancer tumorigenesis (Yu et al., 2020b), and the regulatory effects of miR-107 and TRIAP1 have also been studied (Na et al., 2019; Cai et al., 2020). For example, miR-107 regulates the proliferation and apoptosis of lung cancer cells by targeting TRIAP1. However, the regulatory role of miR-107 and TRIAP1 and the biological role of TRIAP1 in gastric cancer are not yet known. The results of this study show that TRIAP1 is highly expressed as an oncogene in GC. When small interfering RNA is used to knock down the expression of TRIAP1, the proliferation activity of GC cells NCI-N87 is significantly inhibited, and the cell invasion and migration ability is significantly reduced. After using the overexpression vector to induce the overexpression of TRIAP1, the proliferation of NCI-N87 cells was significantly increased, and the cell invasion and migration ability was significantly increased. In addition, dual luciferase reporter gene and rescue experiments confirmed that miR-107 exerts tumor suppressor activity on GC cells NCI-N87 by targeting TRIAP1.

In summary, the results of this study show that miR-107 is lowly expressed as a tumor suppressor gene in GC, and TRIAP1 is highly expressed as an oncogene in GC. The expression of the TRIAP1 and miR-107 is obviously negatively correlated. Further *in vitro* function and mechanism analysis shows that miR-107 inhibits the progress of GC *in vivo* and *in vitro* by targeting TRIAP1. However, this study also has certain limitations, such as the failure to study the correlation analysis between miR-107 expression level and patient survival, because of the sample size. Exploring whether miR-107 can be used as a tumor marker to distinguish GC, and secondly, the downstream signaling pathway of TRIAP1 has not been explored in depth, and this will also be the direction of our later research.

## Data Availability

The raw data supporting the conclusion of this article will be made available by the authors, without undue reservation.
