# Supplementary figures and images for: miR-107 Inhibits the Proliferation of Gastric Cancer Cells In vivo and In vitro by Targeting TRIAP1
Source: Front Genet. 2022 Apr 11;13:855355. doi: 10.3389/fgene.2022.855355 (PMC9035523; doi:10.3389/fgene.2022.855355)

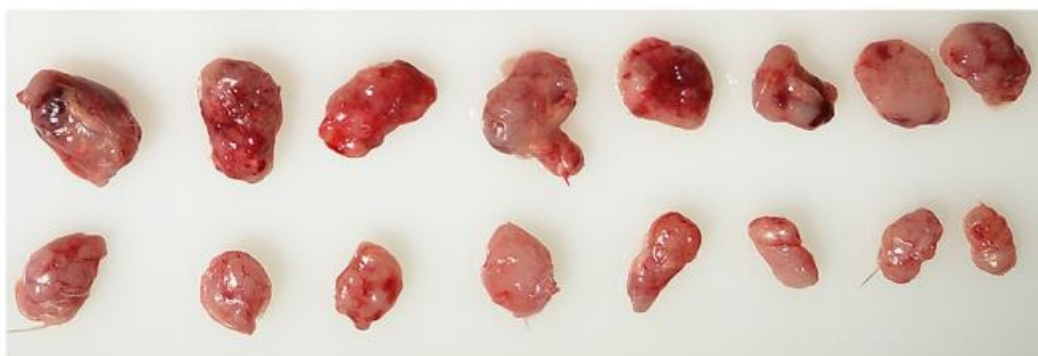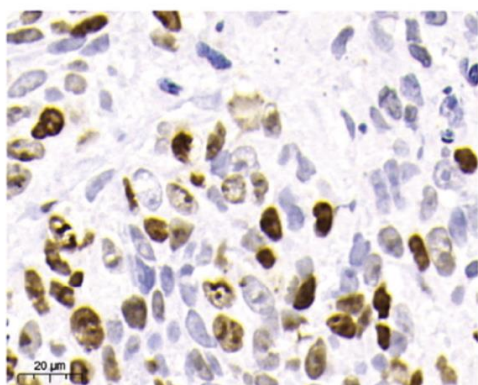

Ki-67

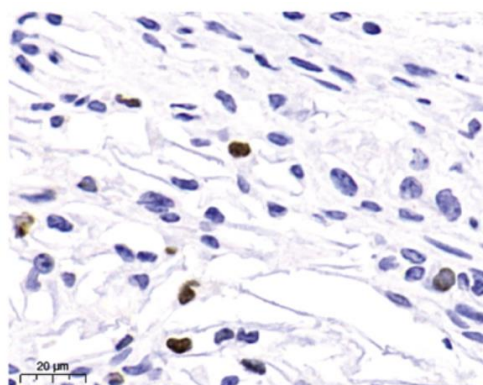

Supplement: Supplementary file 1 [file DataSheet7.PDF]

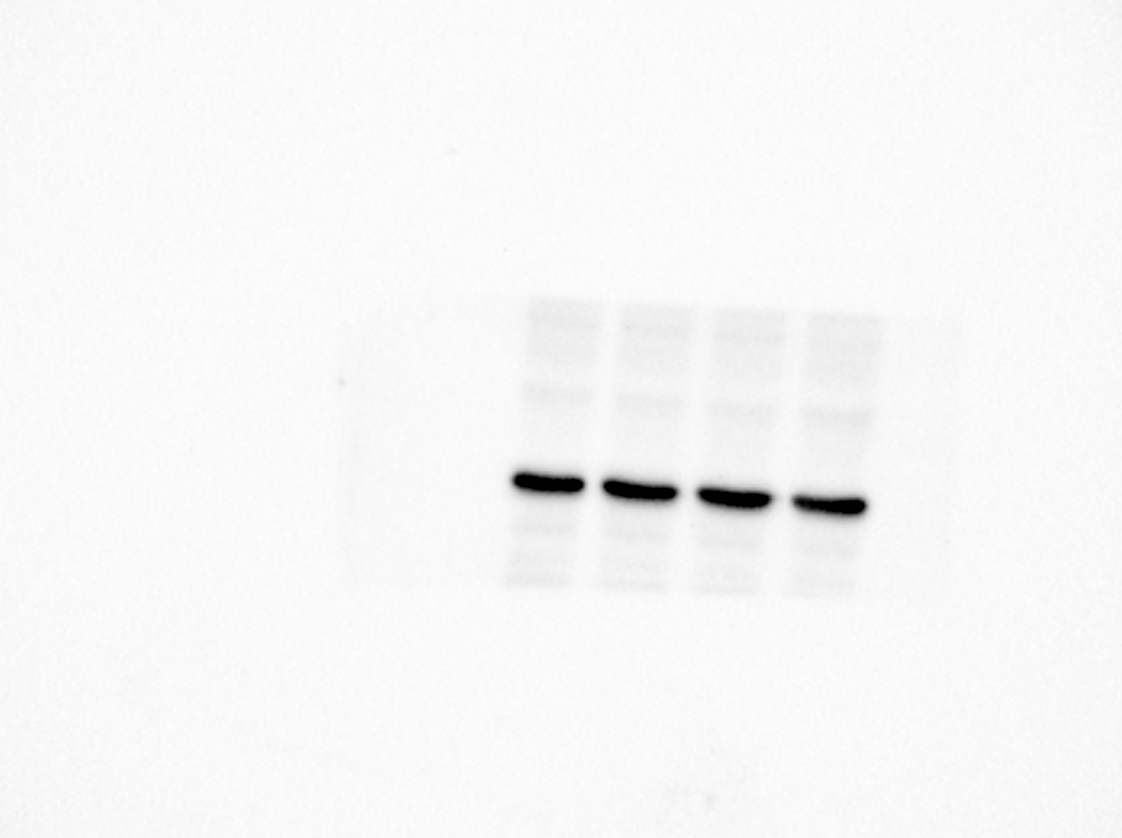

Supplement: Supplementary file 2 [file DataSheet3.ZIP › Western-Blot/Figure 5 GAPDH.tif]

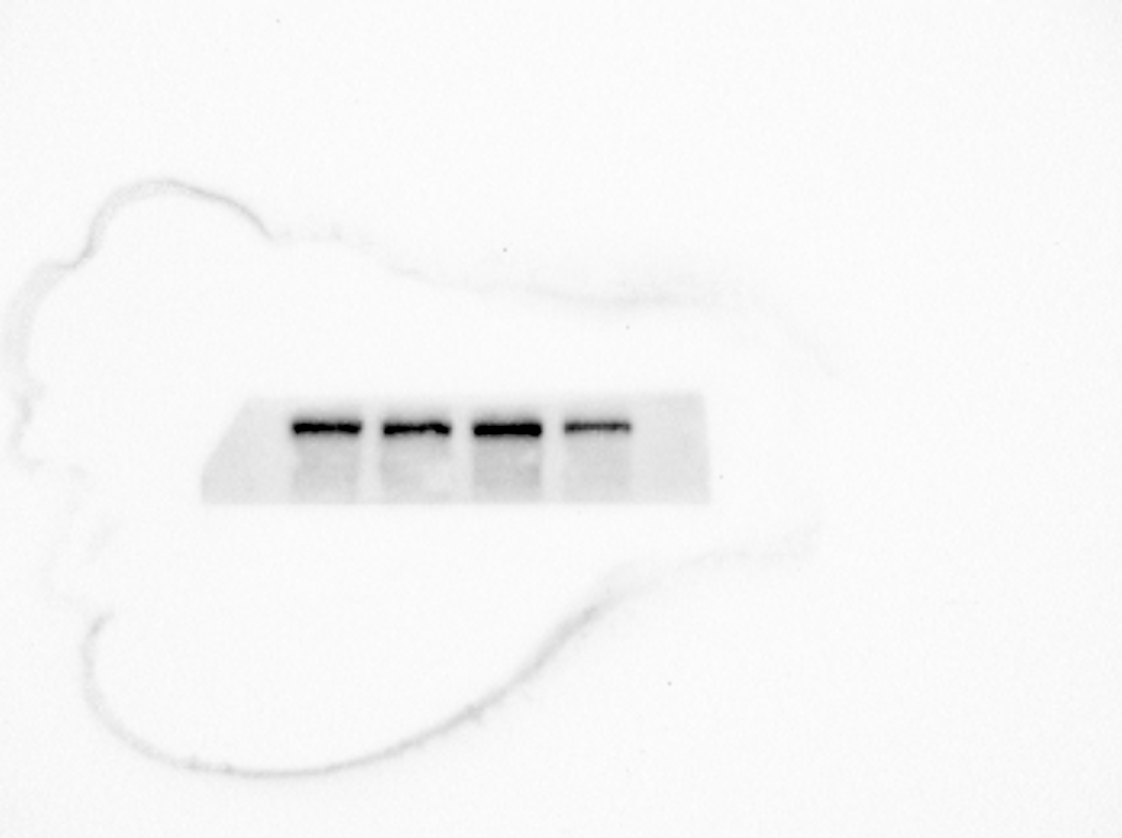

Supplement: Supplementary file 2 [file DataSheet3.ZIP › Western-Blot/Figure 5TRIAP1.tif]

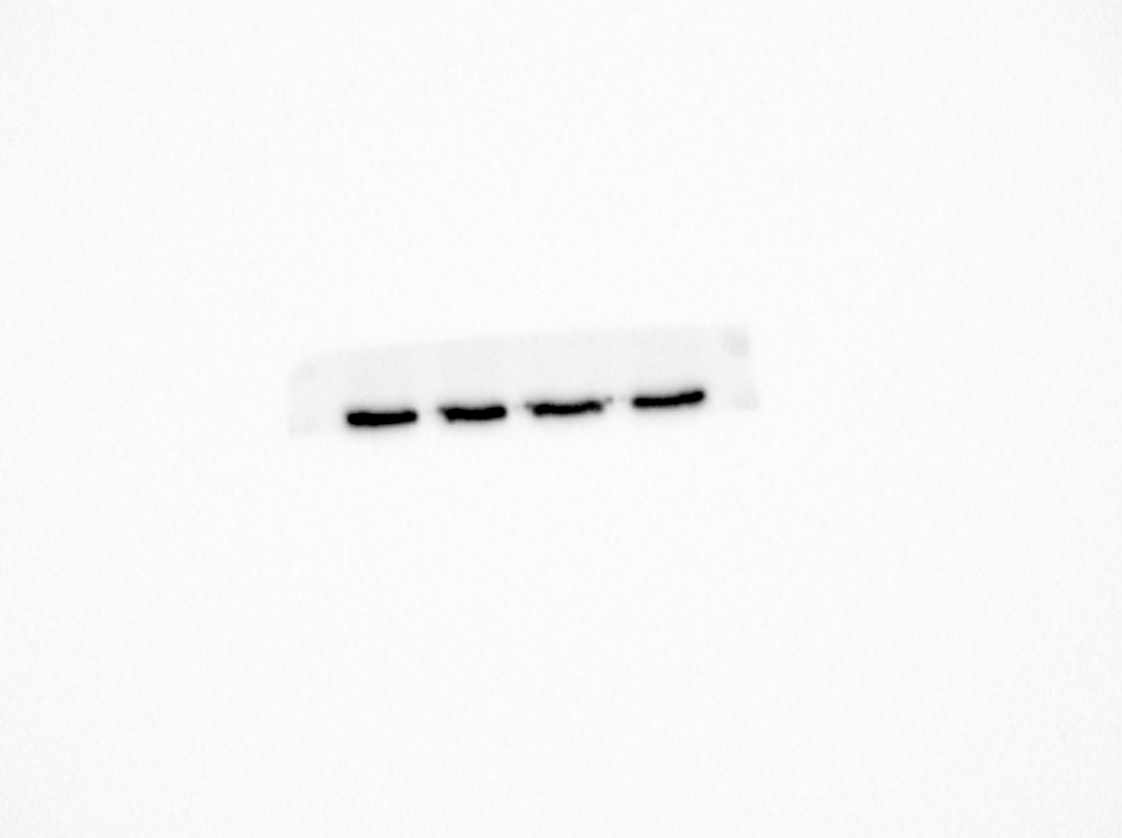

Supplement: Supplementary file 2 [file DataSheet3.ZIP › Western-Blot/Figure1 GAPDH.tif]

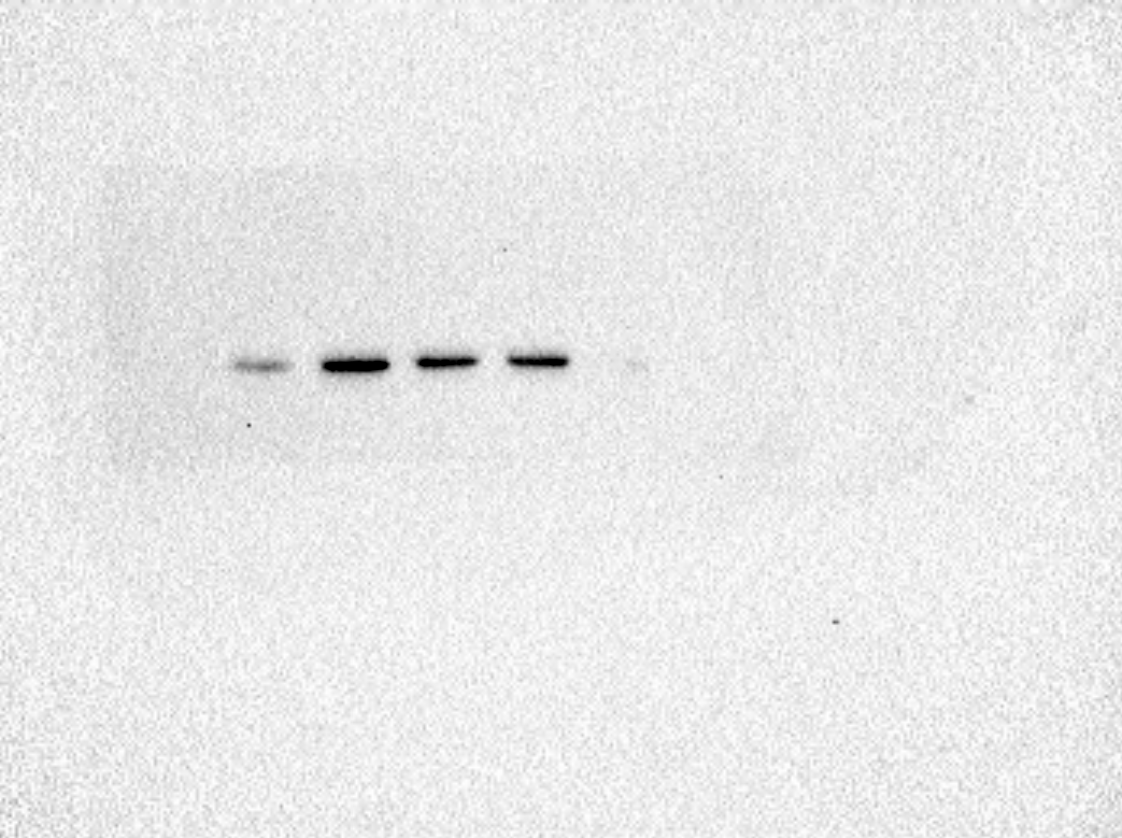

Supplement: Supplementary file 2 [file DataSheet3.ZIP › Western-Blot/figure1 TRIAP1.tif]

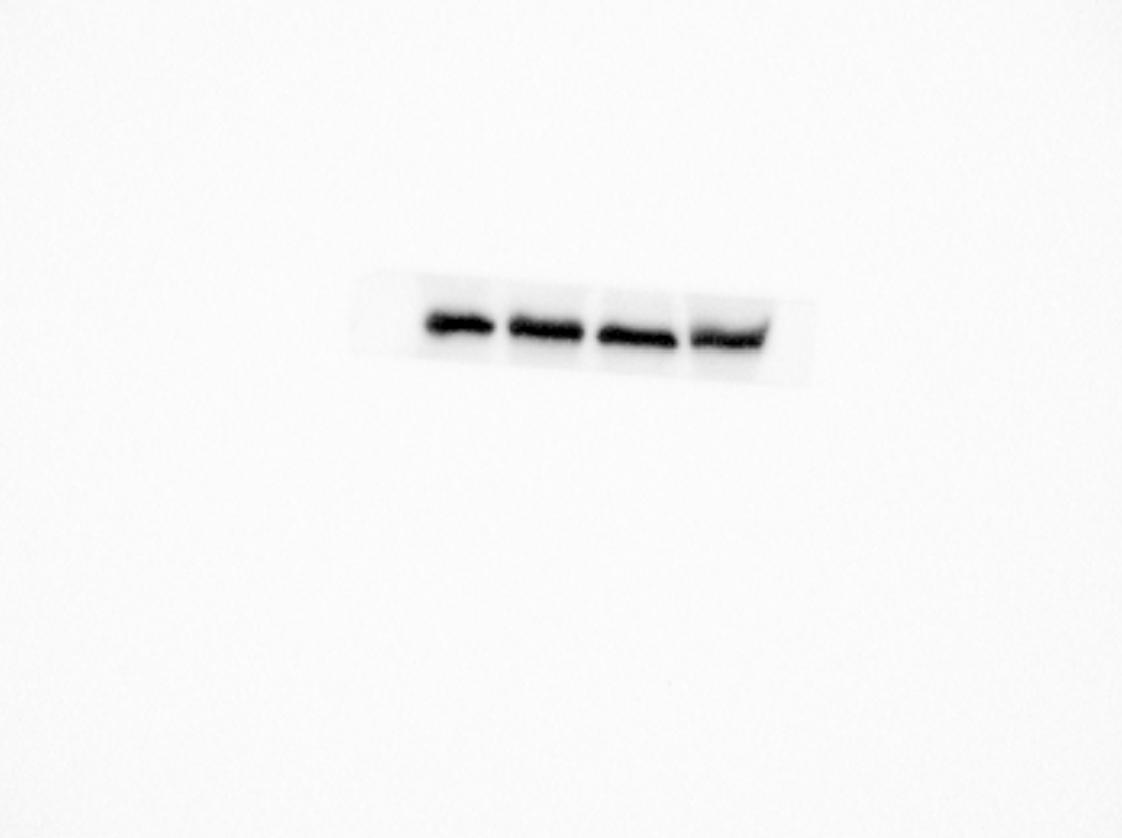

Supplement: Supplementary file 2 [file DataSheet3.ZIP › Western-Blot/figure2 GAPDH.tif]

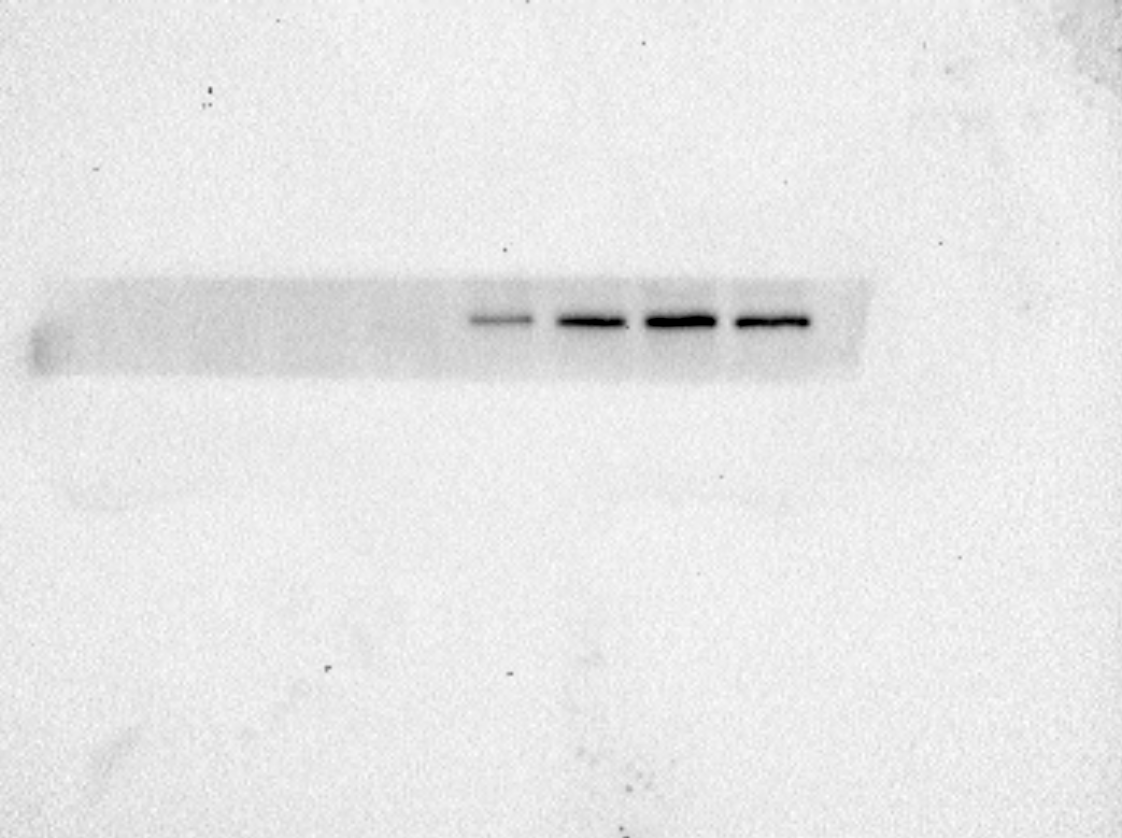

Supplement: Supplementary file 2 [file DataSheet3.ZIP › Western-Blot/figure2 TRIAP1.tif]

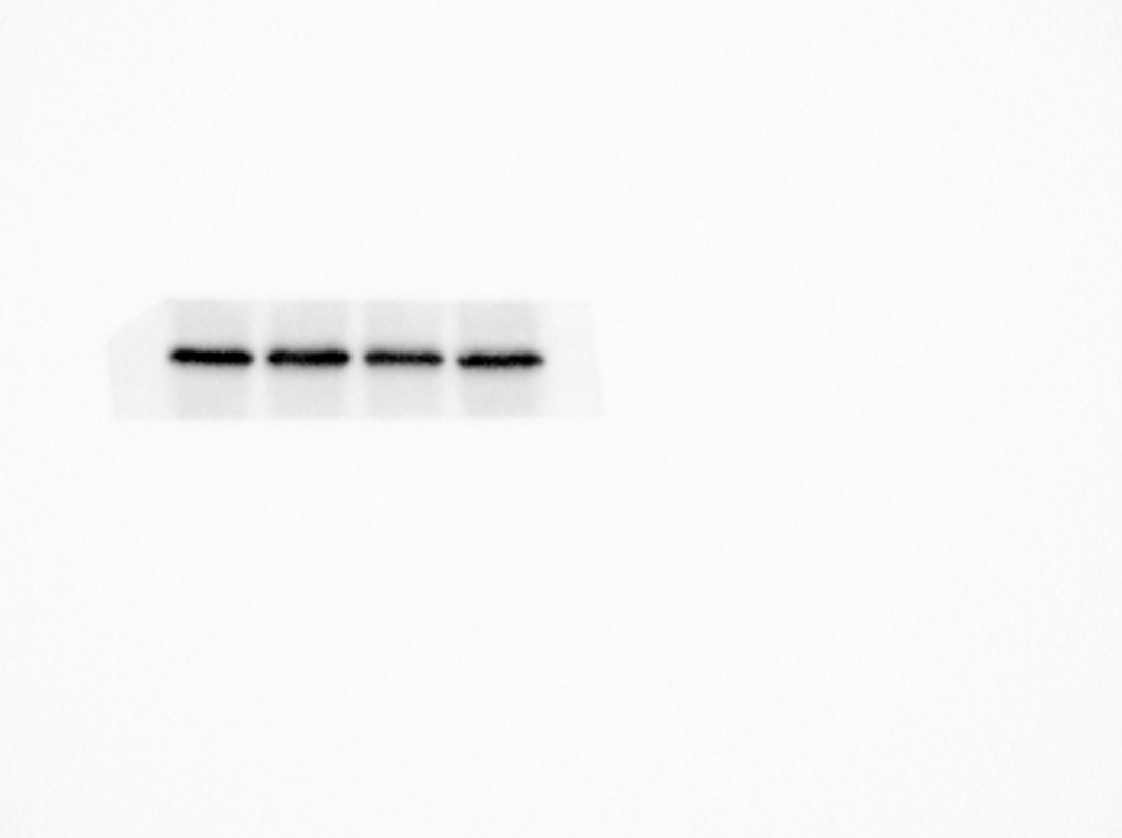

Supplement: Supplementary file 2 [file DataSheet3.ZIP › Western-Blot/Figure3 GAPDH.tif]

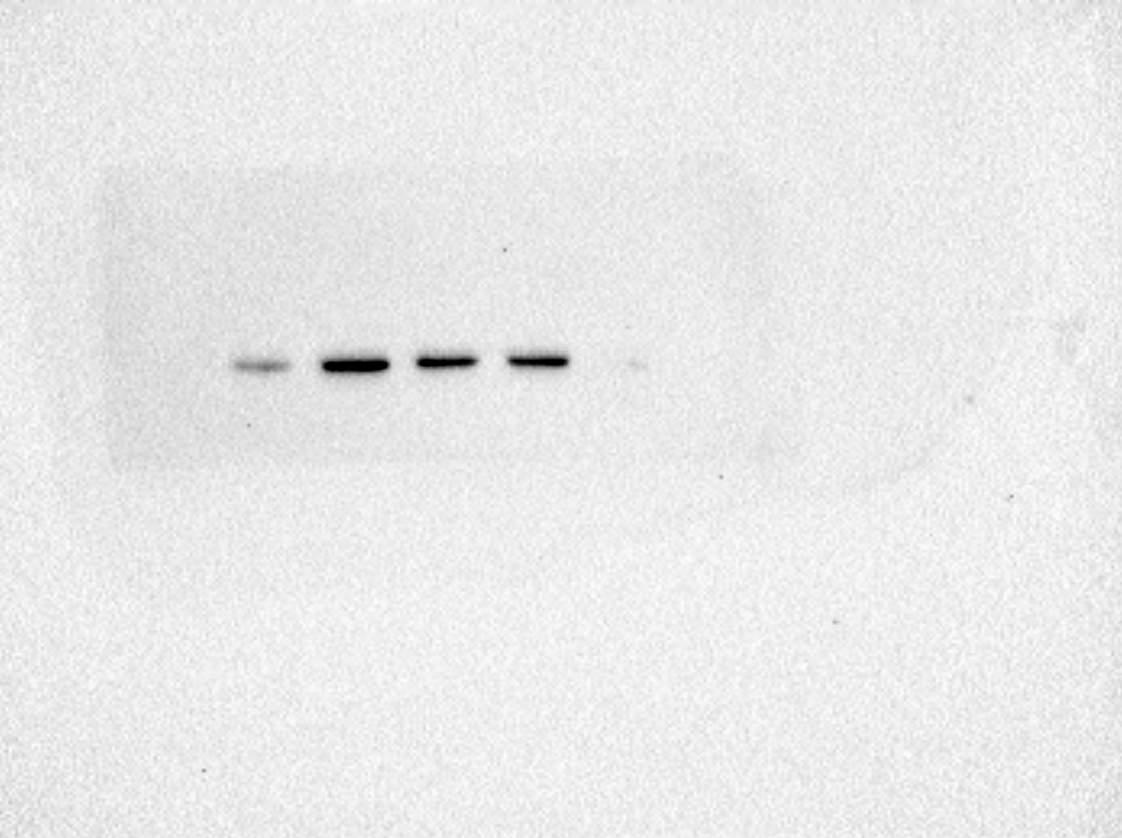

Supplement: Supplementary file 2 [file DataSheet3.ZIP › Western-Blot/Figure3 TRIAP1.tif]

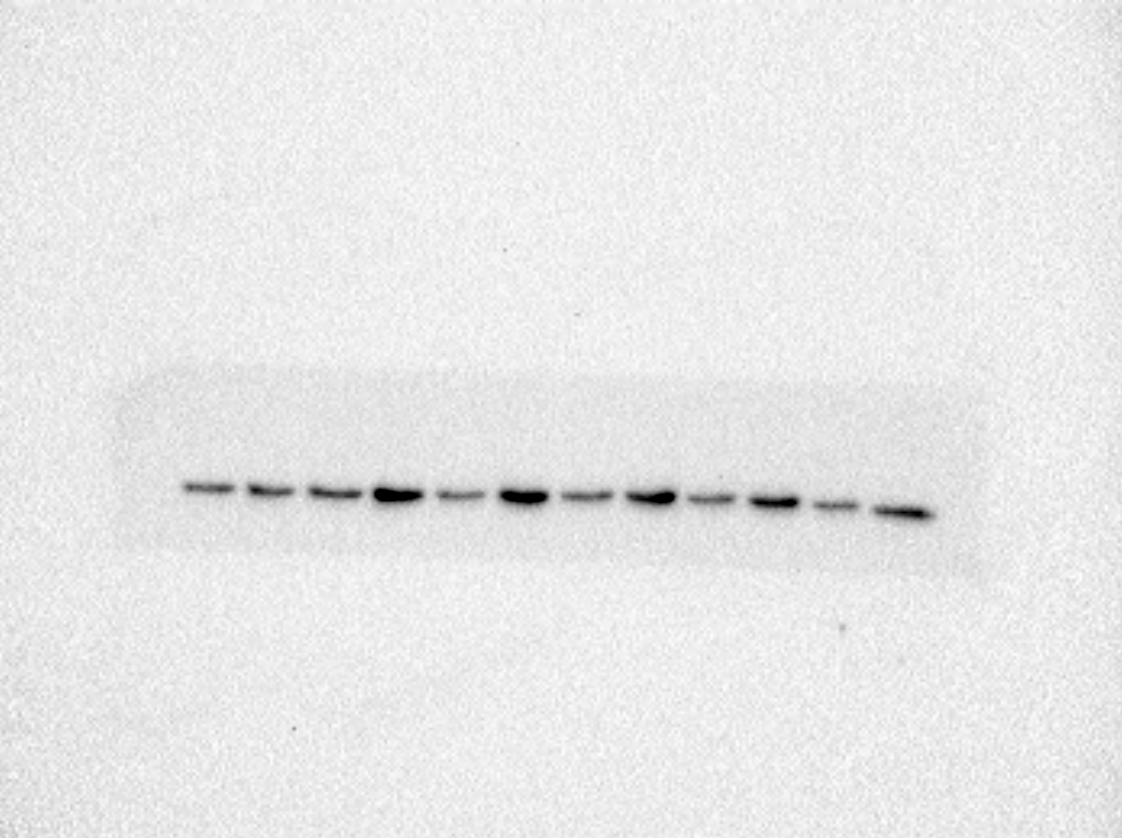

Supplement: Supplementary file 2 [file DataSheet3.ZIP › Western-Blot/Patiens1-6.tif]

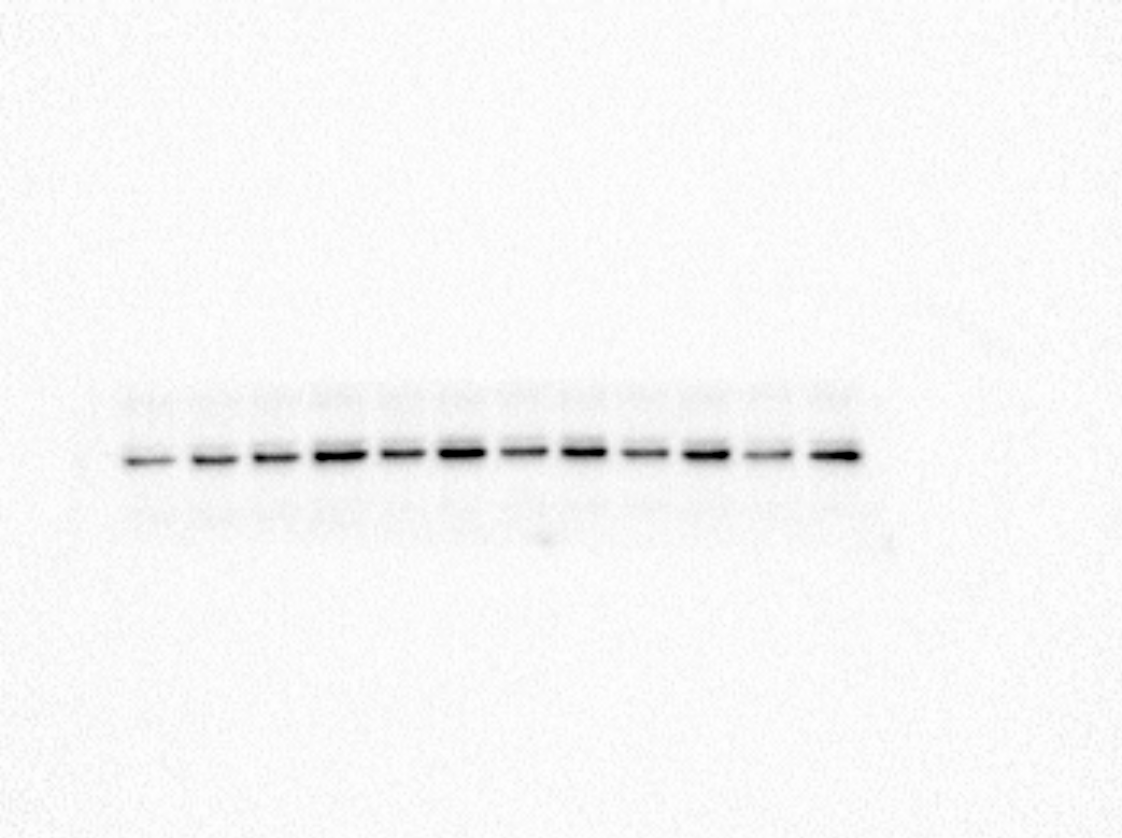

Supplement: Supplementary file 2 [file DataSheet3.ZIP › Western-Blot/Patiens7-12.tif]

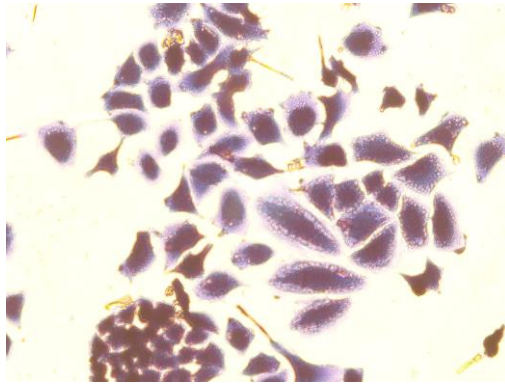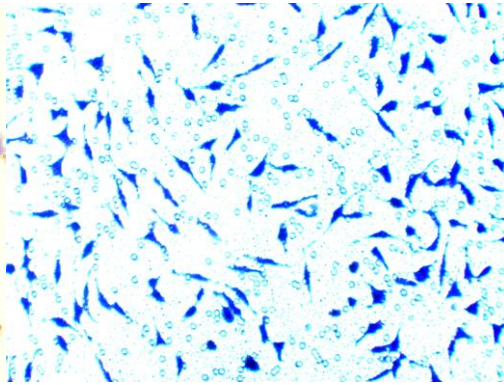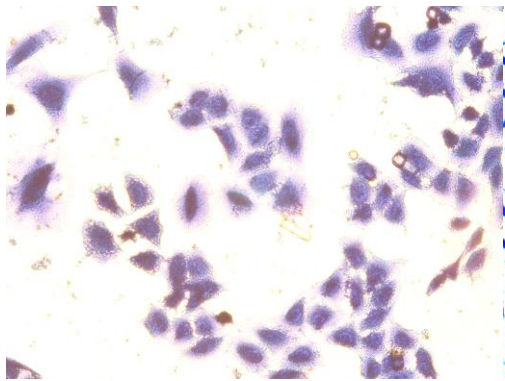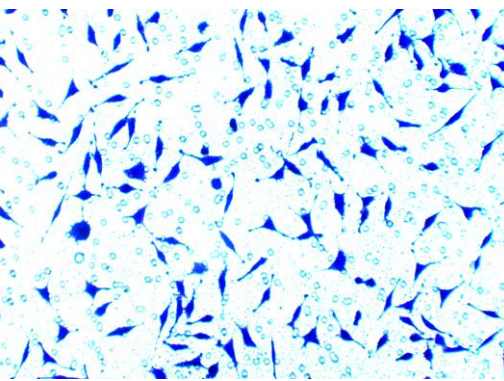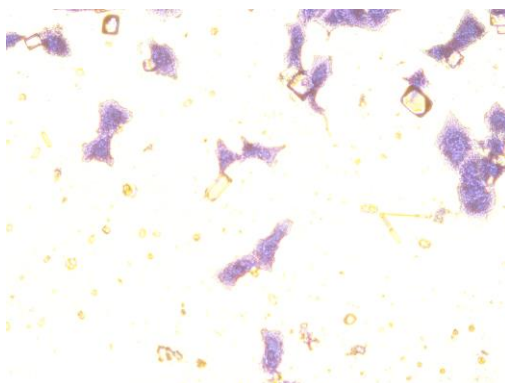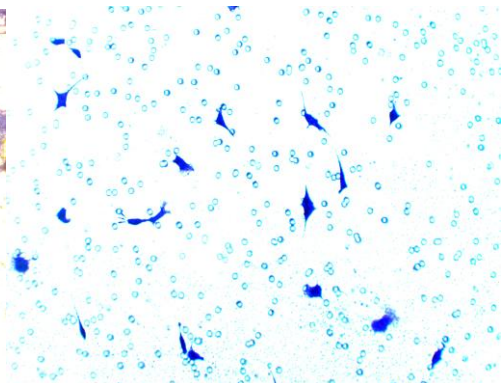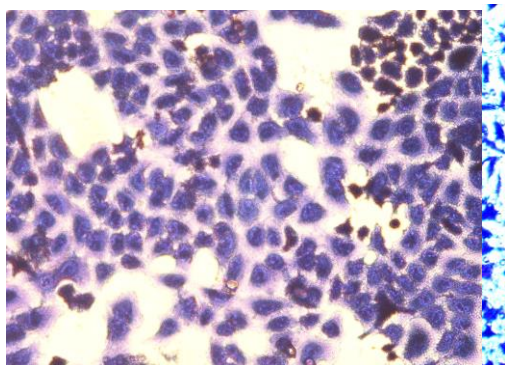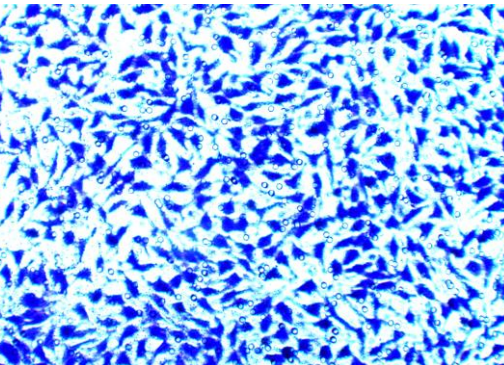

Supplement: Supplementary file 3 [file DataSheet4.PDF]

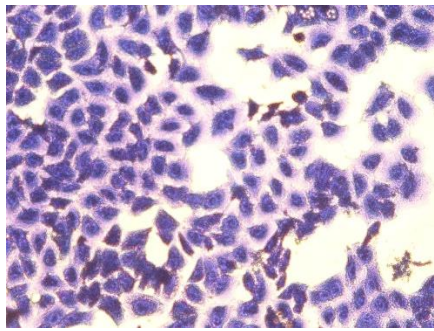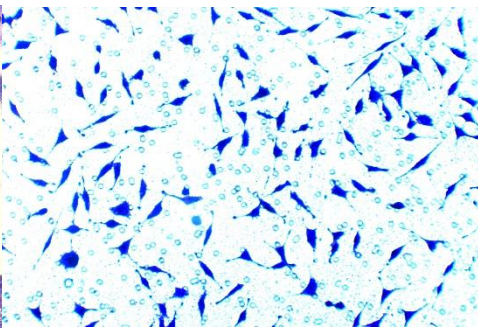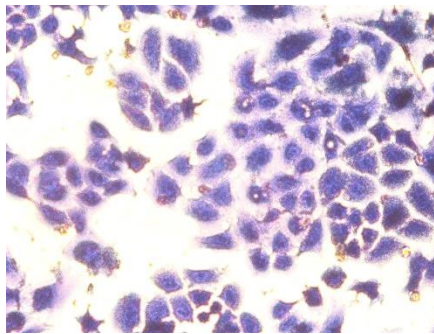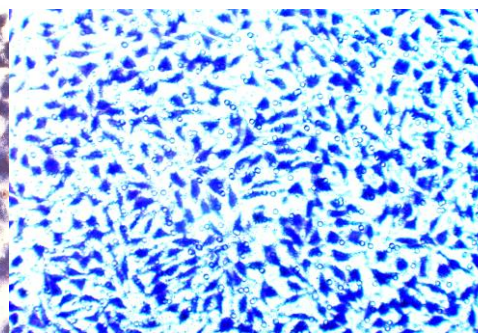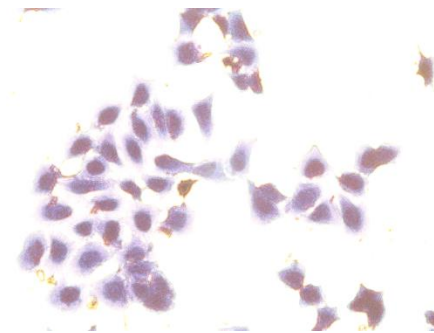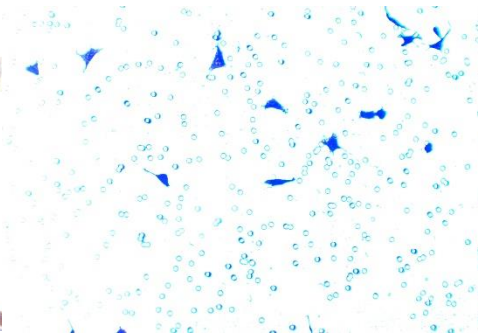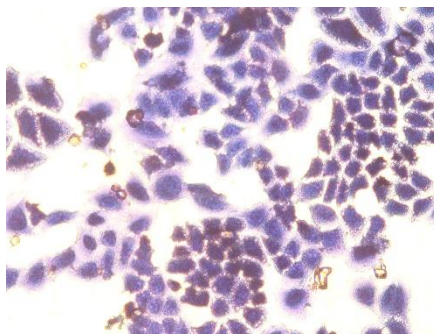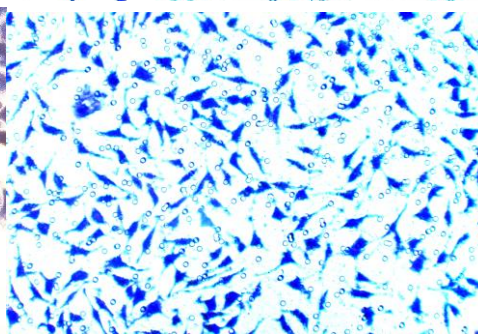

Supplement: Supplementary file 4 [file DataSheet6.PDF]

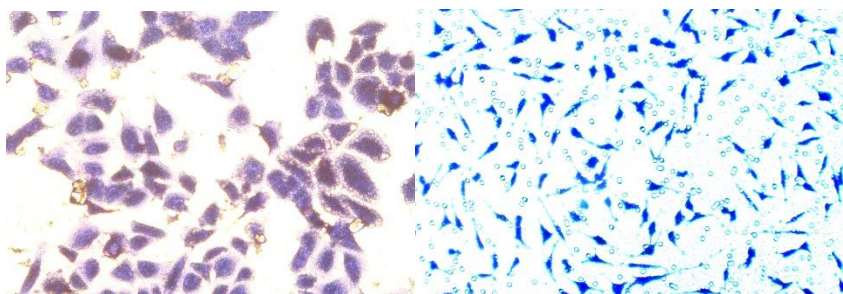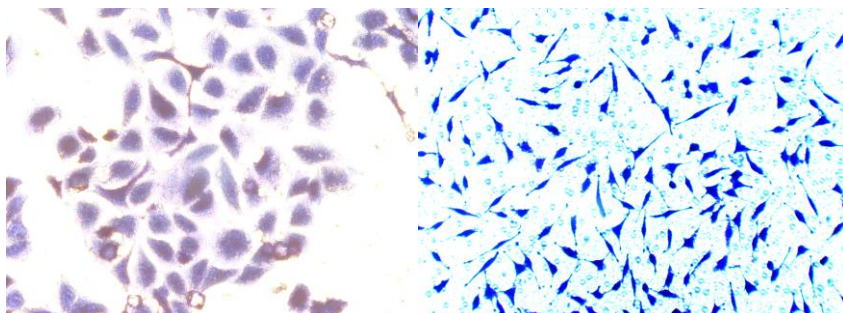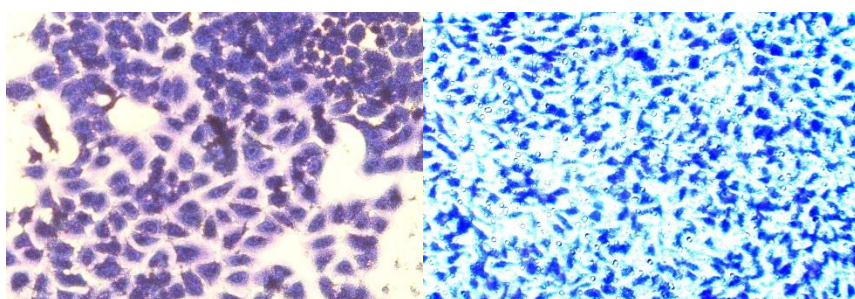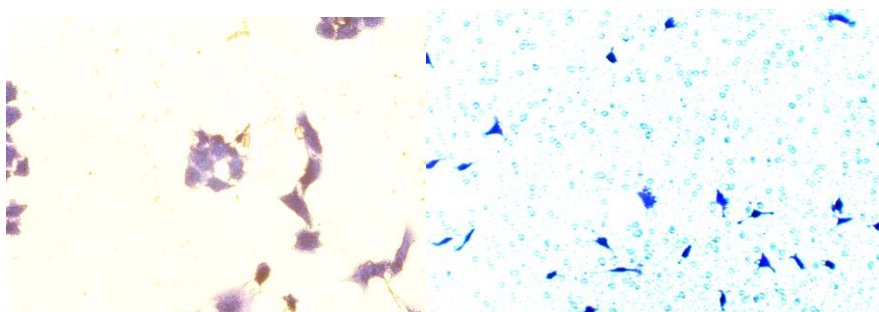

Supplement: Supplementary file 6 [file DataSheet5.PDF]
